# The *cis*‐expression of the coat protein of turnip mosaic virus is essential for viral intercellular movement in plants

**DOI:** 10.1111/mpp.12973

**Published:** 2020-07-19

**Authors:** Zhaoji Dai, Rongrong He, Mark A. Bernards, Aiming Wang

**Affiliations:** ^1^ London Research and Development Centre, Agriculture and Agri‐Food Canada London Ontario Canada; ^2^ Department of Biology The University of Western Ontario London Ontario Canada

**Keywords:** cell‐to‐cell movement, coat protein, potyvirus, protein stability, systemic infection, turnip mosaic virus, virion assembly

## Abstract

To establish infection, plant viruses are evolutionarily empowered with the ability to spread intercellularly. Potyviruses represent the largest group of known plant‐infecting RNA viruses, including many agriculturally important viruses. To better understand intercellular movement of potyviruses, we used turnip mosaic virus (TuMV) as a model and constructed a double‐fluorescent (green and mCherry) protein‐tagged TuMV infectious clone, which allows distinct observation of primary and secondary infected cells. We conducted a series of deletion and mutation analyses to characterize the role of TuMV coat protein (CP) in viral intercellular movement. TuMV CP has 288 amino acids and is composed of three domains: the N‐terminus (amino acids 1–97), the core (amino acids 98–245), and the C‐terminus (amino acids 246–288). We found that deletion of CP or its segments amino acids 51–199, amino acids 200–283, or amino acids 265–274 abolished the ability of TuMV to spread intercellularly but did not affect virus replication. Interestingly, deletion of amino acids 6–50 in the N‐terminus domain resulted in the formation of aberrant virions but did not significantly compromise TuMV cell‐to‐cell and systemic movement. We identified the charged residues R178 and D222 within the core domain that are essential for virion formation and TuMV local and systemic transport in plants. Moreover, we found that *trans*‐expression of the wild‐type CP either by TuMV or through genetic transformation‐based stable expression could not rescue the movement defect of CP mutants. Taken together these results suggest that TuMV CP is not essential for viral genome replication but is indispensable for viral intercellular transport where only the *cis*‐expressed CP is functional.

## Graphical abstract

The turnip mosaic virus coat protein (CP) is not required for viral genome replication but is indispensable for viral cell‐to‐cell and long‐distance movement where only *cis*‐expressed CP is functional.

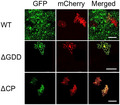

## 1 INTRODUCTION

Viruses are obligate intracellular agents that infect all living organisms and exclusively multiply in their host cells. Viral pathogens account for nearly 50% of newly emerging plant diseases and are considered a major constraint to agriculture, threatening global food security (Anderson *et al*., 2004). The vast majority of known viruses have positive‐sense, single‐stranded (+ss) RNA genomes. To establish systemic infection, a plant virus, regardless of the viral genome being DNA or RNA, must have the ability to move intercellularly from the primary infected cells to neighbouring cells through plasmodesmata (PD), and further undergo long‐distance movement via the phloem and/or xylem to reach remote sites of an infected plant (Folimonova and Tilsner, 2018; Reagan and Burch‐Smith, 2020). PD are specialized intercellular organelles that connect adjacent cells and are gateways to local and systemic infection (Benitez‐Alfonso *et al*., 2010). They allow small molecules to diffuse between cells and regulate the intercellular movement of macromolecules or macromolecular complexes, including virions and viral ribonucleoprotein complexes (vRNPs) (Navarro *et al*., 2019). A better understanding of how an infecting virus manages to move intercellularly to establish systemic infection may assist in the development of novel strategies for disease control. Previous studies have suggested that viral cell‐to‐cell movement is achieved through the coordinated action of virus‐encoded movement proteins (MPs), viral particles and/or vRNPs, and host factors (Schoelz *et al*., 2011; Heinlein, 2015; Wang, 2015; Navarro *et al*., 2019). Typical MPs may be classified into two groups (Navarro *et al*., 2019). The first group, exemplified by the single dedicated 30 kDa MP of tobamoviruses, increases the size exclusion limit of PD, without affecting PD structure, to allow vRNPs to pass through (Kawakami *et al*., 2004; Peña and Heinlein, 2012; Liu and Nelson, 2013). The second group of MPs self‐interacts to form tubular structures that modify the PD pore by replacing the endoplasmic reticulum (ER)‐derived desmotubule and allow the transport of viral particles from the site of virion assembly to neighbouring cells (Ritzenthaler and Hofmann, 2007; Schmitt‐Keichinger *et al*., 2017). Therefore, both MPs and coat proteins (CPs) are required for the viruses with this group of MPs to move intercellularly. MPs of some icosahedral viruses, such as nepo‐ and comoviruses, belong to this group.

Potyviruses represent the largest group of known plant RNA viruses, including many agriculturally important viruses such as *Turnip mosaic virus* (TuMV), *Plum pox virus* (PPV), *Soybean mosaic virus* (SMV), and *Potato virus Y* (PVY) (Revers and García, 2015; Wylie *et al*., 2017; Cui and Wang, 2019; Gibbs *et al*., 2020). Potyviruses have a positive‐sense, single‐stranded RNA genome of approximately 10,000 nucleotides that encodes a long open reading frame (ORF) and additional small ORFs resulting from RNA polymerase slippage during viral genome replication (Olspert *et al*., 2015; Revers and García, 2015; Rodamilans *et al*., 2015; Hagiwara‐Komoda *et al*., 2016; Cui and Wang, 2019). The polyproteins encoded by these ORFs are processed co‐ and post‐translationally into over 10 mature proteins, including P3N‐PIPO and P3N‐ALT. Among them, the viral protein P3N‐PIPO encoded by a small ORF is a dedicated MP (Cui *et al*., 2017). P3N‐PIPO is a PD‐located protein and directs the viral cylindrical inclusion protein (CI) to form conical structures at PD to assist potyviral intercellular movement (Wei *et al*., 2010). In addition to P3N‐PIPO and CI, potyviral CP has also been shown to be essential for potyviral cell‐to‐cell movement (Dolja *et al*., 1994, 1995; Arazi *et al*., 2001; Kimalov *et al*., 2004; Seo *et al*., 2013; Tatineni *et al*., 2014). The potyviral CP encompasses a variable N‐terminal domain exposed on the virion surface that is susceptible to trypsin treatment, a conserved core domain that interacts with viral RNA and forms the core subunit structure of the virion, and a C‐terminal domain that has been implicated in CP–vRNA binding (Zamora *et al*., 2017; Cuesta *et al*., 2019; Kezar *et al*., 2019). Deletion of CP or its important domains or mutation of key charged residues within these domains abolishes potyvirus intercellular movement or systemic infection (Dolja *et al*., 1994, 1995; Seo *et al*., 2013; Kezar *et al*., 2019). The fact that CPs are required for potyviral intercellular movement raises the possibility that potyviral intercellular spread may occur in the form of virions. To date, several studies have been devoted to better understand the mechanisms underlying the requirement of potyviral CPs in viral movement. Some resulting data are inconsistent among potyviruses. For instance, it has been suggested that the N‐terminal region is not required for zucchini yellow mosaic virus (ZYMV) to establish local and systemic infection (Arazi *et al*., 2001). However, deletion of this region from PVY and tobacco etch virus (TEV) reduces viral local infectivity and abolishes viral systemic infection (Dolja *et al*., 1994; Kezar *et al*., 2019). In the case of *Wheat streak mosaic virus* (WSMV), a member of the genus *Tritimovirus* in the family *Potyviridae*, this region is not essential for long‐distance movement but is required for efficient cell‐to‐cell movement (Tatineni and French, 2014; Tatineni *et al*., 2014). In addition, a few other studies have reported that the potyviral replication complex (VRC) can be found in the extracellular space or move to the adjacent cells (Grangeon *et al*., 2012, 2013; Movahed *et al*., 2019), implying that potyviruses may move intercellularly in the form of vRNPs.

In this study, we constructed a double‐fluorescent protein‐tagged TuMV infectious clone that can unambiguously distinguish primary and secondary infected cells to investigate the role of TuMV CP in viral intercellular movement. We confirmed that TuMV CP is not required for viral replication but is indispensable for viral cell‐to‐cell movement and systemic infection in plants. We further identified the regions and charged amino acids of TuMV CP that are required for TuMV to establish local and systemic infection. We present evidence that CP expressed in *trans* cannot rescue the movement defect of TuMV CP mutants, suggesting that the *cis*‐expression of CP is essential for TuMV intercellular movement.

## 2 RESULTS

### 2.1 TuMV CP is indispensable for viral cell‐to‐cell and systemic movement but not required for viral replication

**Figure 1:**
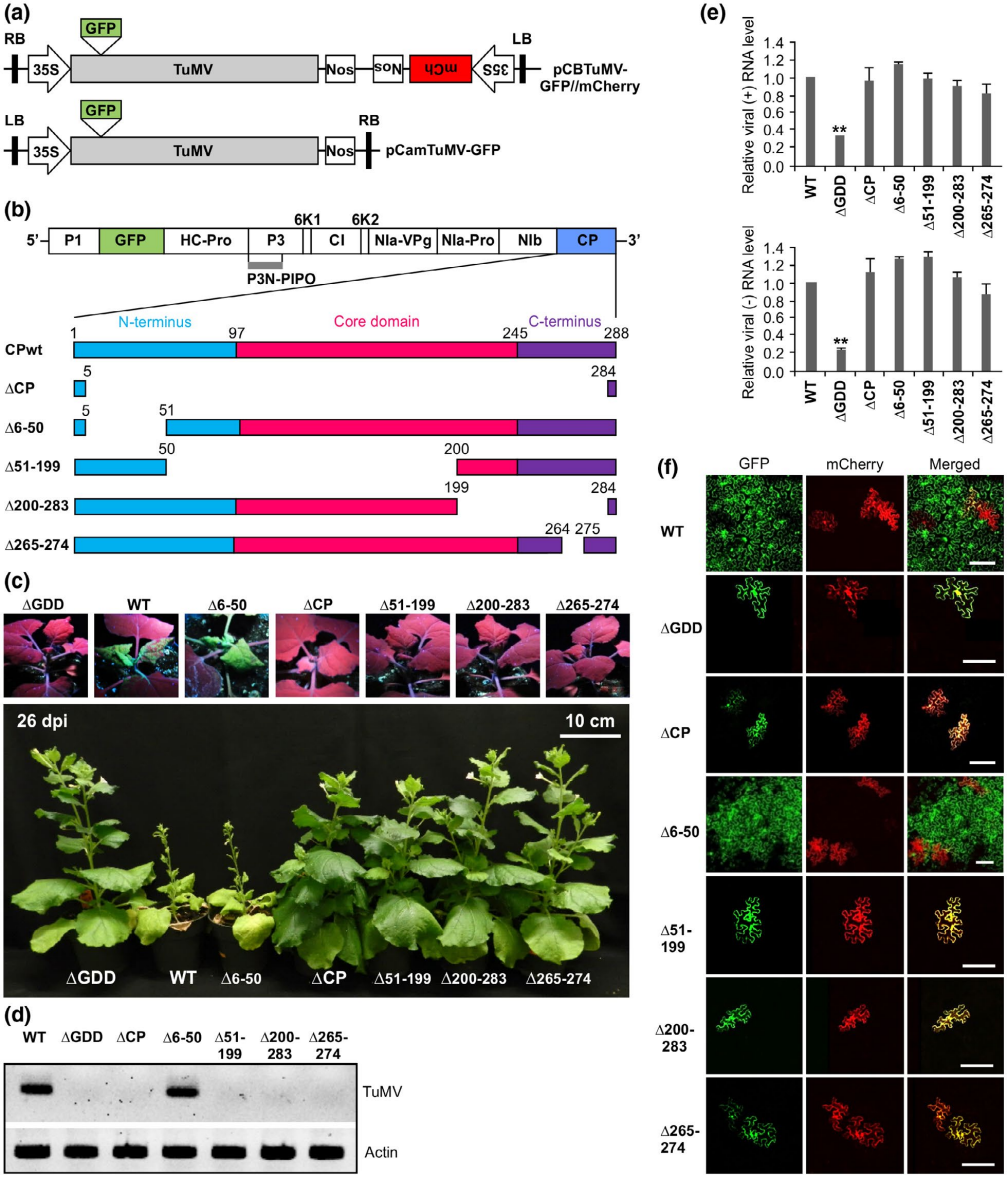
Effects of coat protein (CP) deletion mutations on TuMV replication, cell‐to‐cell movement, and systemic infection in *Nicotiana benthamiana*. (a) Schematic representation of TuMV infectious clones used in this study. Upper panel: Schematic representation of the infectious clone pCBTuMV‐GFP//mCherry for distinguishing between primary and secondary infection sites. Bottom panel: Schematic representation of the infectious clone pCamTuMV‐GFP for TuMV replication assay in protoplasts. mCh, mCherry tagged by the endoplasmic reticulum (ER) retention signal HDEL. (b) Schematic representation of wild‐type (WT) TuMV and CP deletion mutants. Green fluorescent protein (GFP) was inserted between P1 and HC‐Pro cistrons of the TuMV genome. (c) Analysis of systemic infection of CP deletion mutants. ΔGDD is a replication‐defective mutant that serves as a negative control. Top panels: visualization of GFP fluorescence in the upper new leaves of *N. benthamiana* plants inoculated under UV light at 9 days postinoculation (dpi). Bottom panel: photograph of the representative *N. benthamiana* plants inoculated with TuMV WT and mutants at 26 dpi. (d) Reverse transcription (RT)‐PCR analysis of viral RNA from systemic leaf of mutants‐inoculated plants at 14 dpi. (e) Replication analysis of CP deletion mutants in protoplasts. Total RNA was extracted from *N. benthamiana* protoplasts transfected with TuMV WT or mutants at 48 hours post‐transfection and viral (+)‐strand RNA (top panel) or (−)‐strand RNA (bottom panel) were quantified by quantitative RT‐PCR. Error bars represent the standard deviation of three biological replicates. **p < .001. (f) Confocal microscopy analysis of cell‐to‐cell movement of CP deletion mutants at 4 dpi. Scale bar = 100 µm

To unambiguously determine viral cell‐to‐cell movement, we constructed a double‐fluorescent (green and mCherry) protein‐tagged TuMV infectious clone, which is designated pCBTuMV‐GFP//mCherry and serves as a wild‐type (WT) virus for this study (Figure 1a). In this vector, two gene expression cassettes are placed within the transfer DNA (T‐DNA) borders: one for transcription of mRNA coding for mCherry fused with a luminal endoplasmic reticulum (ER) retention signal (mCherry‐HDEL) and the other for the TuMV genome tagged by green fluorescent protein (GFP). On agroinfiltration of this clone into *Nicotiana benthamiana* leaf cells, both GFP and mCherry fluorescent proteins are expected to be expressed in the primary infected cells, leading to the emission of green and red fluorescence signals. The secondary infected cells as a result of viral intercellular movement would emit green fluorescence only as the recombinant TuMV genome contains the GFP sequence. After confirming that this WT clone allows for the differential visualization of primary and secondary infected cells (see below), we used it as a parental plasmid and constructed two additional clones. The first, ∆GDD, has a deletion in the coding sequence for the glycine‐aspartic acid‐aspartic acid (GDD) motif that is the active site of the RNA‐dependent RNA polymerase (also NIb) (Shen *et al*., 2020). The second, ∆CP, was created by the deletion of almost the entire coding region for the CP except two short stretches essential for the cleavage sites (Figure 1b). ∆GDD is a replication‐defective mutant and serves as a control (Deng *et al*., 2015) (Figure 1c–f). These three constructs, WT, ∆GDD, and ∆CP, were transformed into *Agrobacterium tumefaciens* GV3101 and then agroinfiltrated into *N. benthamiana* leaf cells at a low OD_600_ value of 0.0001. As expected, the WT virus systemically infected the *N. benthamiana* plants by 9 days postinoculation (dpi) and green fluorescence was clearly observed in the upper new leaves under UV light (Figure 1c). Confocal microscopy of the WT‐infiltrated leaf areas at 4 dpi detected isolated individual cells emitting both red and green fluorescence and also clustered cells emitting green fluorescence only (Figure 1f). Both ∆GDD and ∆CP lost infectivity as no green fluorescence was evident in the upper new leaves under UV light at 9 dpi or over an extended period (26 dpi) of observation (Figure 1c) and reverse transcription‐polymerase chain reaction (RT‐PCR) failed to detect the virus in the upper new leaves (Figure 1d). In agroinfiltrated regions at 4 dpi, only isolated individual cells emitting both the mCherry and GFP fluorescent signals were found under a confocal microscope (Figure 1f), suggesting no viral intercellular movement occurred for these two mutants. Potyviral cell‐to‐cell movement requires active genome replication so that a small percentage of viruses encoding P3N‐PIPO, a dedicated movement protein, is generated (Cui *et al*., 2017). As the ∆GDD mutant lost cell‐to‐cell movement ability, the expression of double fluorescent proteins in agroinfiltrated cells with ∆GDD could be due to the 35S promoter activity.

To determine if deletion of CP compromises viral replication leading to the inability to move between cells, we isolated mesophyll protoplasts from 4‐week‐old healthy *N. benthamiana* seedlings and conducted a protoplast transfection assay with ∆CP. Quantitative RT‐PCR (RT‐qPCR) analyses revealed that the level of either viral plus‐strand or negative‐strand RNA in ∆CP‐transfected protoplasts 48 hrs post‐transfection (hpt) did not significantly differ from that in WT‐transfected protoplasts but was significantly higher than that in the protoplasts transfected with ∆GDD (Figure 1e). Taken together these data suggest that TuMV CP is essential for viral intercellular and systemic movement but is not required for viral replication.

### 2.2 Identification of CP segments required for viral cell‐to‐cell movement

The TuMV CP comprises 288 amino acid residues with a molecular mass of approximately 33 kDa. Based on the recently released atomic model (PDB: 6T34) (Cuesta *et al*., 2019), TuMV CP is divided into the N‐terminus (N, amino acids 1–97), core (amino acids 98–245), and C‐terminus (C, amino acids 246–288) domains (Figure 1b). To identify the CP segments that are essential for TuMV cell‐to‐cell movement, we created four partial CP deletion mutants using the parental WT virus (pCBTuMV‐GFP//mCherry) (Figure 1b and Table 2). Deletion of the segment amino acids 6–50 located in the N (Δ6–50) did not obviously affect viral infectivity as the *N. benthamiana* plants agroinfiltrated with Δ6–50 excited strong GFP signals in leaves distal to the infiltrated leaf under UV light and exhibited mosaic and stunting symptoms, similar to the plants agroinfiltrated with the WT (Figure 1c). In contrast, plants agroinfiltrated with any of the remaining three mutants Δ51–199, Δ200–283, and Δ265–274 did not develop any obvious symptoms, and under UV light the upper new leaves of the plants did not show detectable GFP signals (Figure 1c). Apparently, these three partial CP deletion mutants failed to establish systemic infection. Total RNA was extracted from the upper new leaves of the plants agroinfiltrated with all CP mutants and controls at 14 dpi, and then analysed by RT‐PCR with TuMV‐specific primers. Consistently, viral RNA was detected from the Δ6–50 sample but not from the Δ51–199, Δ200–283, or Δ265–274 samples (Figure 1d). Next, we conducted a protoplast transfection assay to check the replication capacity of these deletion mutants in the *N. benthamiana* protoplasts. At 48 hpt, the viral RNA levels of CP deletion mutants were comparable to that of the WT (Figure 1e). These data further confirm that CP is dispensable for TuMV replication.

We then examined the cell‐to‐cell movement ability of the mutants. The *N. benthamiana* leaves agroinfiltrated with each of these mutants were subjected to confocal microscopy at 4 dpi. In leaf tissues agroinfiltrated with the mutant ∆6–50, we found isolated individual cells emitting double fluorescent signals and also large foci of green fluorescence, similar to what was observed for the WT (Figure 1f). However, in the agroinfiltrated leaf regions with Δ51–199, Δ200–283, or Δ265–274, we observed isolated cells highlighted by double fluorescence but did not detect any cells emitting green fluorescence only (Figure 1f). These data suggest that similar to ∆CP, mutants Δ51–199, Δ200–283, and Δ265–274 lose the ability to move from the primary infected cells to neighbouring cells.

### 2.3 Deletion of the segment amino acids 6–50 of the N domain results in the production of aberrant viral particles

**Figure 2:**
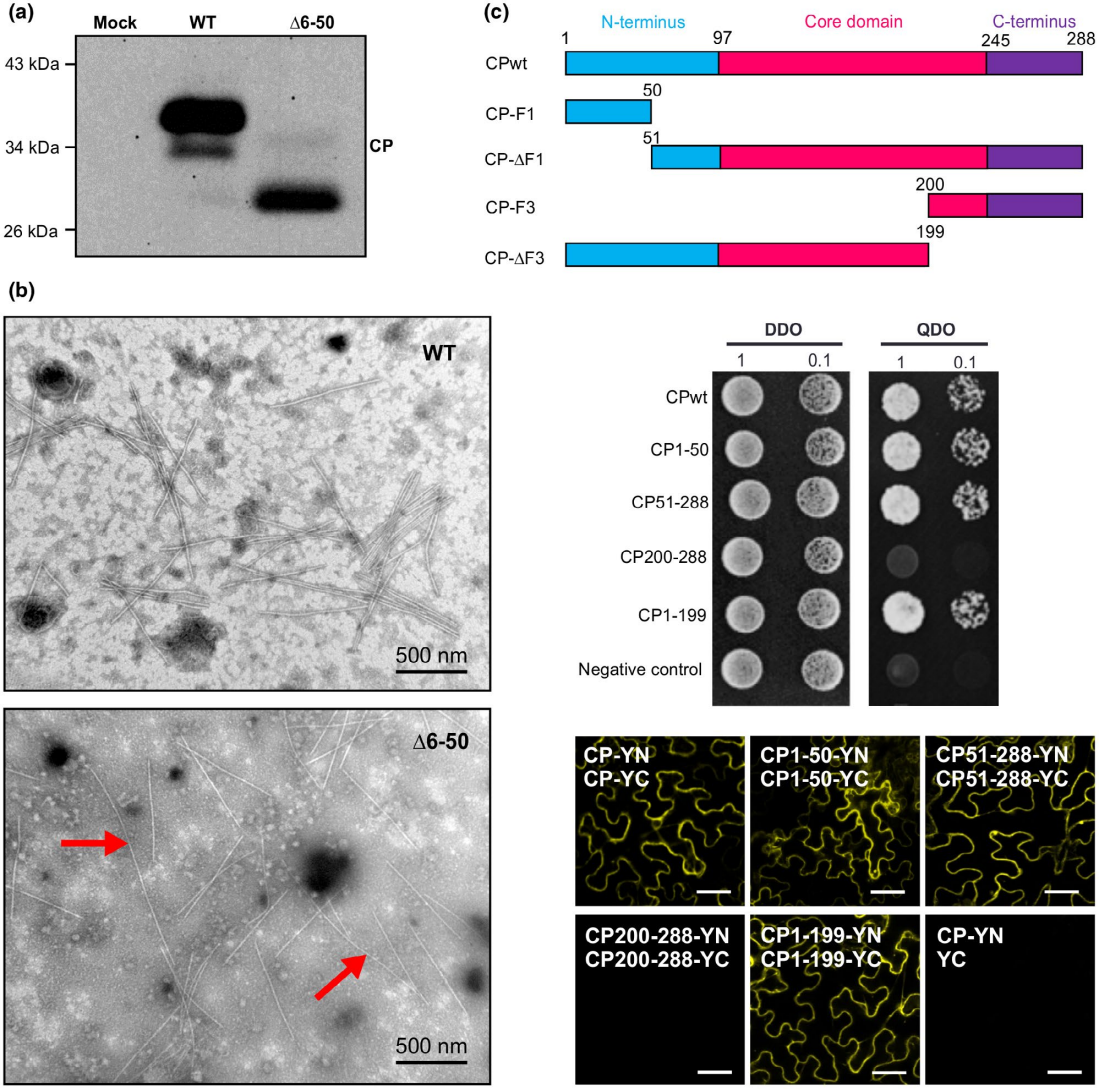
Effects of deletion of a coat protein (CP) segment (amino acids 6–50) on virion assembly and analysis of the self‐interactions of different CP regions. (a) Immunoblotting analysis of total proteins extracted from TuMV wild‐type (WT) or Δ6–50‐infected plants at 12 days postinoculation (dpi). Total protein extracts were probed with TuMV CP antibody. (b) Transmission electron microscopy (TEM) analysis of virion assembly of Δ6–50 mutant. Crude virion preparations were obtained from the symptomatic tissue of WT or Δ6–50‐infected plants at 12 dpi. The preparation was subjected to negative staining and TEM. (c) Analysis of the self‐interactions of different CP regions. Top panel: schematic representation of truncated portions for protein–protein interaction assays. Middle panel: yeast two‐hybrid (Y2H) analysis in yeast. Yeast competent cells co‐transformed with bait and prey plasmids were plated on double dropout (DDO) medium lacking tryptophan and leucine to test for double transformation, and on quadruple dropout (QDO) medium lacking tryptophan, leucine, histidine, and adenine for protein–protein interaction. Yeast co‐transformed with AD‐CP and BD‐CP serves as a positive control. Yeast co‐transformed with AD‐CP and empty BD plasmid serves as a negative control. Bottom panel: bimolecular fluorescence complementation analysis of CP‐truncated mutants in *Nicotiana benthamiana* plants. Combination of CP‐YN/CP‐YC serves as a positive control and CP‐YN/YC as the negative control. Experiments were repeated three times. Bars = 40 µm

Because the N‐terminal region of some potyviral CPs such as PPV is known to be involved in the CP–CP interaction, which is important for virion assembly (Zilian and Maiss, 2011), we determined whether ∆6–50 can form regular virions in plant cells. Crude virus extracts were prepared from *N. benthamiana* leaves systemically infected by WT or ∆6–50 at 12 dpi. Immunoblotting analyses with polyclonal antibodies to TuMV CP detected a protein of slightly larger than 33 kDa from the WT sample and a protein of about 28 kDa from the ∆6–50 sample, consistent with the predicted molecular mass for WT CP and ∆6–50, respectively (Figure 2a). The crude virus extracts were also subjected to negative staining and transmission electron microscopy (TEM) analysis. Numerous typical flexuous rod‐shaped viral particles of 600–900 nm in length were easily observed in the WT sample (Figure 2b). In addition to the typical TuMV particles as shown in the WT sample, a small percentage of particles in the ∆6–50 sample had remarkably elongated length (Figure 2b). To quantify the distribution of aberrant particles in the ∆N samples, we measured 120 particles (randomly selected) and found 5% of particles showed the elongation phenotype with an average length of 1,500 nm. No such elongated particles were observed in the WT sample. These findings suggest that though the N‐terminal segment amino acids 6–50 of the N domain is probably involved in TuMV virion assembly, deletion of this region is not sufficient to completely disrupt the formation of morphologically typical viral particles.

Because self‐interaction is required for virion assembly, we generated four truncated CP mutants (Figure 2c) and examined their ability to self‐interact using both yeast two‐hybrid (Y2H) and bimolecular fluorescence complementation (BiFC) assays. Y2H results revealed that CP200‐288 did not self‐interact, whereas CPwt, CP1–50, CP1–199, and CP51–288 did (Figure 2c). Consistent results were obtained with the BiFC assay in planta (Figure 2c).These data suggest that the C domain is unlikely to be involved in virion assembly through self‐interaction whereas other regions probably are.

### 2.4 Identification of residues essential for TuMV infection

**Figure 3:**
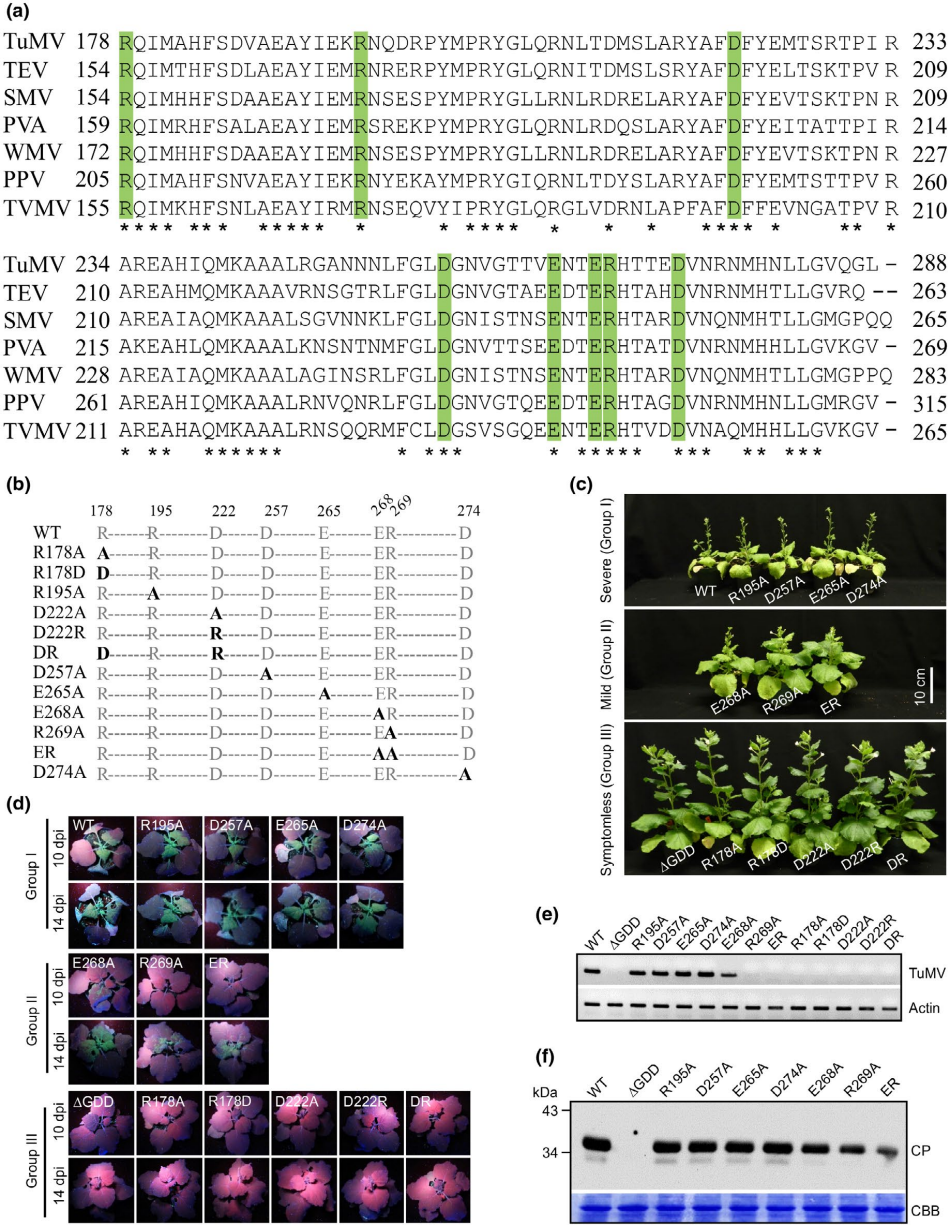
Effects of coat protein (CP) point mutations on TuMV systemic infection. (a) Protein sequence alignment of CP derived from different potyviruses. The abbreviated species names and their GenBank accession numbers are as follows: TuMV, *Turnip mosaic virus* (NC_002509); TEV, *Turnip etch virus* (NC_001555); SMV, *Soybean mosaic virus* (FJ807700); PVA, *Potato virus A* (NC_004039); WMV, *Watermelon mosaic virus* (NC_006262); PPV, *Plum pox virus* (NC_001445); TVMV, *Tobacco vein mottling virus* (NC_001768). Asterisks indicate identical residues and amino acids subjected to substitution are coloured in green. (b) Schematic representation of the point mutations on TuMV CP. (c) Phenotypes of *Nicotiana benthamiana* plants inoculated with TuMV wild‐type (WT) and mutants at 26 days postinoculation (dpi). (d) Analysis of systemic infection of point mutants by visualization of green fluorescent protein (GFP) fluorescence under UV light at 10 and 14 dpi. (e) Reverse transcription‐PCR analysis of viral RNA from the upper new leaves of plants in (c) at 10 dpi. TuMV‐specific primers for the CP coding region were used to detect viral positive‐strand RNA. The Actin gene serves as an internal control. (f) Immunoblotting analysis of point mutants that can establish systemic infection in *N. benthamiana* plants. Total protein extracts from the upper leaf at 14 dpi were probed with TuMV CP antibody. The Coomassie brilliant blue (CBB)‐stained RuBisCO large subunit serves as a loading control

Given the fact that the CP fragment encompassing the C‐terminal segment of the core domain and the entire C domain lacks self‐interaction ability (Figure 2c) but is essential for viral intercellular moment (Figure 1f), we conducted a protein sequence alignment analysis on this fragment from TuMV and six additional commonly studied potyviruses (Figure 3a). We found that this region shared 50% amino acid identity. Among the conserved residues, we selected eight charged ones for alanine substitution analyses. We generated a total of 12 single‐ or double‐point mutation mutants: R178A, R178D, R195A, D222A, D222R, DR (R178D/D222R), D257A, E265A, E268A, R269A, ER (E268A/R269A), and D274A (Figure 3b and Table 2).

To test their infectivity, these single‐ or double‐point mutation mutants were agroinfiltrated into 4‐week‐old *N. benthamiana* leaves. Viral infection in these plants was monitored by visual observation of induced symptoms, visualization of green fluorescence under UV light, and RT‐PCR to detect the virus. Based on the symptoms observed at 26 dpi, the 12 mutants were divided into three groups: I, the WT‐like virus group consisting of R195A, D257A, E265A, and D274A, which induced severe symptoms as the WT; II, the mild virus group consisting of E268A, R269A, and ER, which caused milder symptoms than the WT; and III, the defective virus group consisting of R178A, R178D, D222A, D222R, and DR, which failed to induce detectable symptoms (Figure 3c).

At 10 dpi, green fluorescence was clearly observed in the upper leaves of the plants agroinfiltrated with the group I mutants and the WT virus under UV light (Figure 3d). At this time point, the virus was detected in the upper leaves of almost all plants inoculated (Table 1). At 26 dpi, the inoculated plants developed symptoms such as severe mottling and distorted leaves, and a stunted plant stature (Figure 3c). We also monitored the CP accumulation level in the upper new leaves at 14 dpi. This group of mutants accumulated a similar level of CP to the WT virus (Figure 3f).

**Table 1:** Effect of TuMV coat protein (CP) point mutations on systemic infection of *Nicotiana benthamiana*

Inoculum	Infectivity^a^		
	10 dpi (%)	14 dpi (%)	26 dpi (%)
WT	24/24 (100)	24/24 (100)	24/24 (100)
GDD	0/24 (0)	0/24 (0)	0/24 (0)
R195A	24/24 (100)	24/24 (100)	24/24 (100)
D257A	23/24 (95.8)	24/24 (100)	24/24 (100)
E265A	24/24 (100)	24/24 (100)	24/24 (100)
D274A	24/24 (100)	24/24 (100)	24/24 (100)
E268A	18/24 (75.0)	24/24 (100)	24/24 (100)
R269A	11/24 (45.8)	20/24 (83.3)	22/24 (91.7)
ER	12/24 (50.0)	21/24 (87.5)	23/24 (95.8)
R178A	0/24 (0)	0/24 (0)	0/24 (0)
R178D	0/24 (0)	0/24 (0)	0/24 (0)
D222A	0/24 (0)	0/24 (0)	0/24 (0)
D222R	0/24 (0)	0/24 (0)	0/24 (0)
DR	0/24 (0)	0/24 (0)	0/24 (0)

^a^ Infectivity is defined as the number of systemically infected plants/number of inoculated plants. Leaves were examined by symptom appearance, UV light, and reverse transcription‐PCR using TuMV CP‐specific primers. Results of four trials were combined. WT, wild type; dpi, days postinoculation.

The group II mutants E268A, R269A, and ER were apparently less infective than the group I and WT viruses. In the upper leaves of the plants infiltrated with the group II mutants, fluorescence was not observed until 10–14 dpi and the signals were much weaker (Figure 3d). Consistently, the onset of symptoms in the infected plants was delayed. Mild symptoms such as some mosaic leaves and slightly reduced plant size were observed in the plants infiltrated with the group II mutants at 26 dpi (Figure 3c). At 14 dpi, this group of mutants accumulated reduced levels of CP in the upper leaves in comparison with the WT and group I viruses (Figure 3f).

Remarkably, the group III mutants R178A, R178D, D222A, D222R, and DR failed to induce any visible symptoms in the infiltrated plants (Figure 3c). In the upper leaves of the infiltrated plants, no green fluorescence could be seen under UV light and RT‐PCR failed to detect the TuMV viral RNA (Figure 3d,e). Obviously, the group III mutants lost the ability to move systemically.

### 2.5 Compromised viral infectivity of CP point mutants is attributed to deficient cell‐to‐cell movement

**Figure 4:**
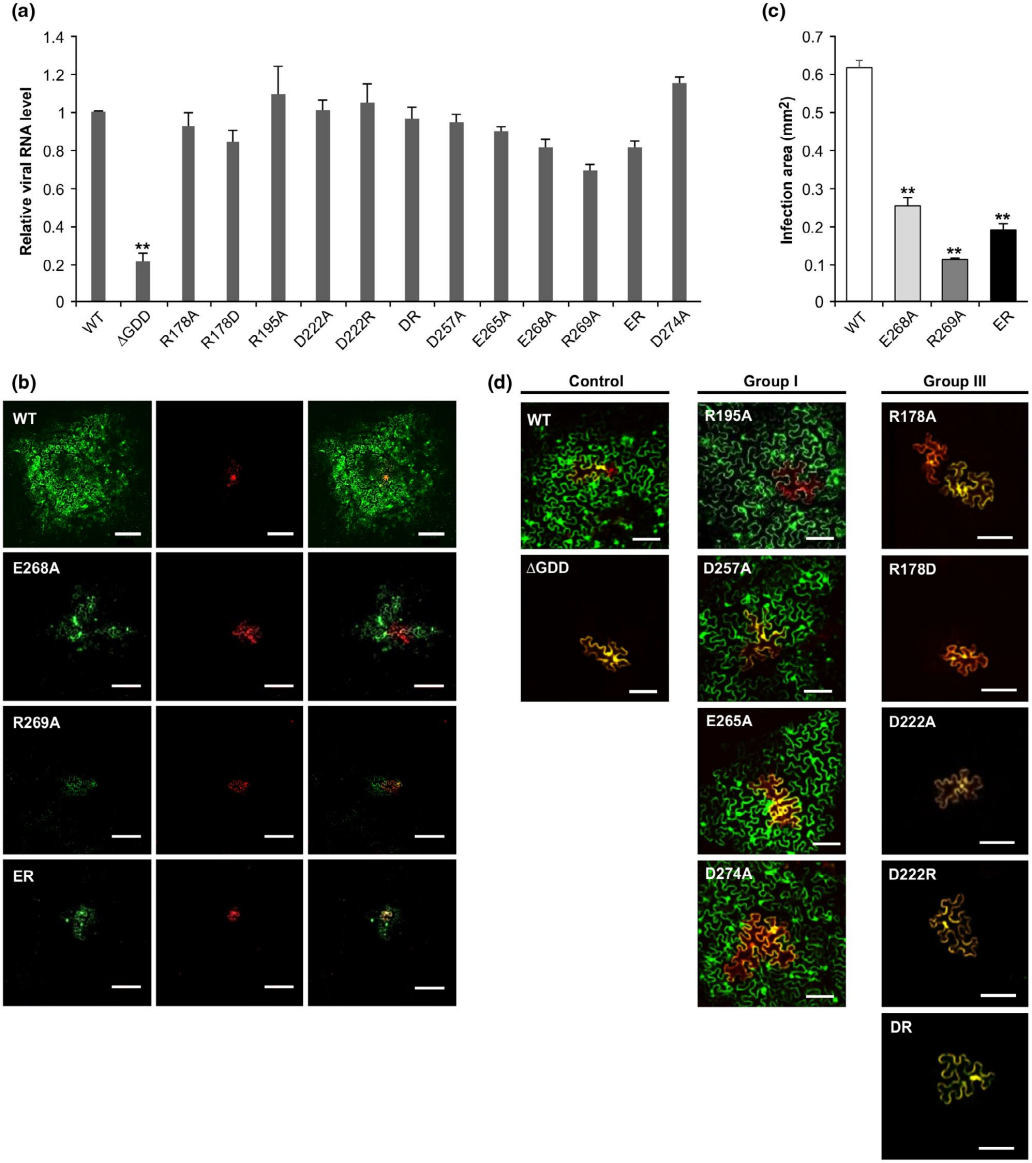
Analysis of replication and cell‐to‐cell movement of coat protein (CP) point mutants. (a) Replication analysis of CP point mutants in protoplasts. Total RNA was extracted from wild‐type (WT) or mutants‐transfected protoplasts at 48 hours post‐transfection and viral (+)‐strand RNA was quantified by quantitative reverse transcription PCR. Error bars represent the *SD* of three biological replicates. **p < .001. (b) Cell‐to‐cell movement analysis of group II mutants. Confocal images were taken from infiltrated leaves at 4 days postinoculation (dpi). Scale bar = 200 µm. (c) Infection foci sizes on *Nicotiana benthamiana* leaves inoculated with the WT virus or group II mutants. Green fluorescent areas were measured under a confocal microscope at 4 dpi. Areas are in square millimetres ± *SD* estimated by ImageJ software. Error bars represent the *SD* of three biological replicates. **p < .001. (d) Cell‐to‐cell movement analysis of group I and group III mutants. Confocal images were taken from infiltrated leaves at 4 dpi. Scale bar = 100 µm

To exclude the possibility that viral replication is affected in the CP point mutants, we conducted a protoplast transfection assay. The plus‐strand viral RNA level was quantified by RT‐qPCR at 48 hpt. All CP point mutants replicated well as their viral RNA levels showed no significant difference with that of the WT virus (Figure 4a).

We then checked the ability of these mutants to move from the primary infected cells to the neighbouring cells by confocal microscopy. Four‐week‐old *N. benthamiana* seedlings were agroinfiltrated at an OD_600_ value of 0.0001. At 4 dpi, in the WT‐infiltrated leaf tissue, both the primary infected cell highlighted by dual fluorescence and a number of surrounding cells emitting green fluorescence only were detected (Figure 4b), indicating a strong ability of the WT to move between cells. The group I mutants that established regular systemic infection (Figure 3c,d) spread like the WT (Figure 4d). In contrast, in the leaf tissues infiltrated with the group II mutants, only a few cells emitted green fluorescence (Figure 4b). The average area of infection foci of the group II mutants was significantly smaller than that of the WT (Figure 4c). These results suggest that the delayed onset of systemic infection of the group II mutants is associated with their slow intercellular spread.

Different from the group I and II mutants, the group III mutants failed to move between cells and showed the phenotype of abolished cell‐to‐cell movement as none of cells emitting green fluorescence only could be found (Figure 4d). This explains why the group III virus completely lost the ability to establish systemic infection.

### 2.6 Effects of CP point mutations on virus assembly

**Figure 5:**
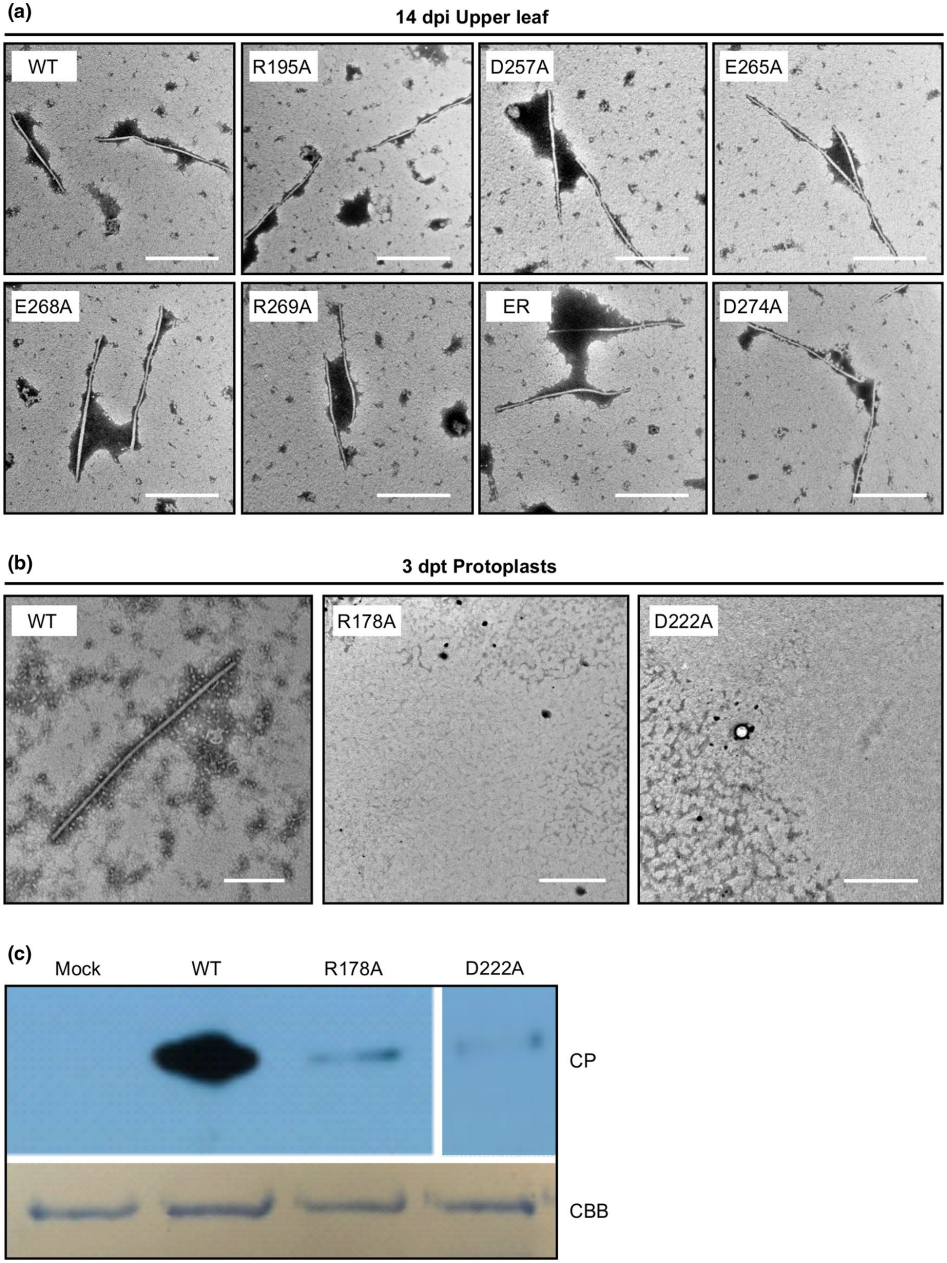
Effects of coat protein (CP) point mutations on virion assembly. (a) Transmission electron microscopy (TEM) analysis of virion assembly of group I and group II mutants. Crude virion preparations were obtained from the upper new leaves of the mutant‐infected *Nicotiana benthamiana* at 14 days postinoculation (dpi). Scale bar = 500 nm. (b) TEM analysis of virion preparations from *N. benthamiana* protoplasts transfected with wild‐type (WT) TuMV, R178A, or D222A mutants at 72 hours post‐transfection (hpt). Scale bar = 200 nm. (c) Immunoblotting analysis of *N. benthamiana* protoplasts transfected with WT, R178A, or D222A. Total protein extracts from protoplasts were probed with TuMV CP antibody. Coomassie brilliant blue staining (CBB) of RuBisCO large subunit serves as a loading control

As potyviral cell‐to‐cell movement is often correlated with virion assembly (Dolja *et al*., 1994, 1995; Seo *et al*., 2013), we examined if the TuMV movement‐defective CP mutants could assemble morphologically normal virions. We collected the upper symtomatic leaves of *N. benthamiana* plants agroinfiltrated with WT, group I or II mutants at 14 dpi, and conducted TEM using crude virion preparations from these samples. As shown in Figure 5a, both the group I and II mutants yielded flexuous and filamentous virions, typical of WT potyviruses.

Because the group III mutants lost the ability to move between cells, we transfected R178A, D222A, and WT into *N. benthamiana* mesophyll protoplasts. The transfected protoplasts were subjected to immunoblotting and TEM analyses. As expected, the CP protein and typical virions were detected from the sample prepared from the protoplasts transfected with WT (Figure 5b,c). However, no virions were found from the protoplasts transfected with R178A and D222A (Figure 5b) and the CP level of these two mutants was remarkably low compared to that of WT (Figure 5c). These data suggest that the cell‐to‐cell movement‐defective mutants R178A and D222A cannot form regular viral particles and their CPs are probably not stable.

### 2.7 CP stability is essential for cell‐to‐cell movement

**Figure 6:**
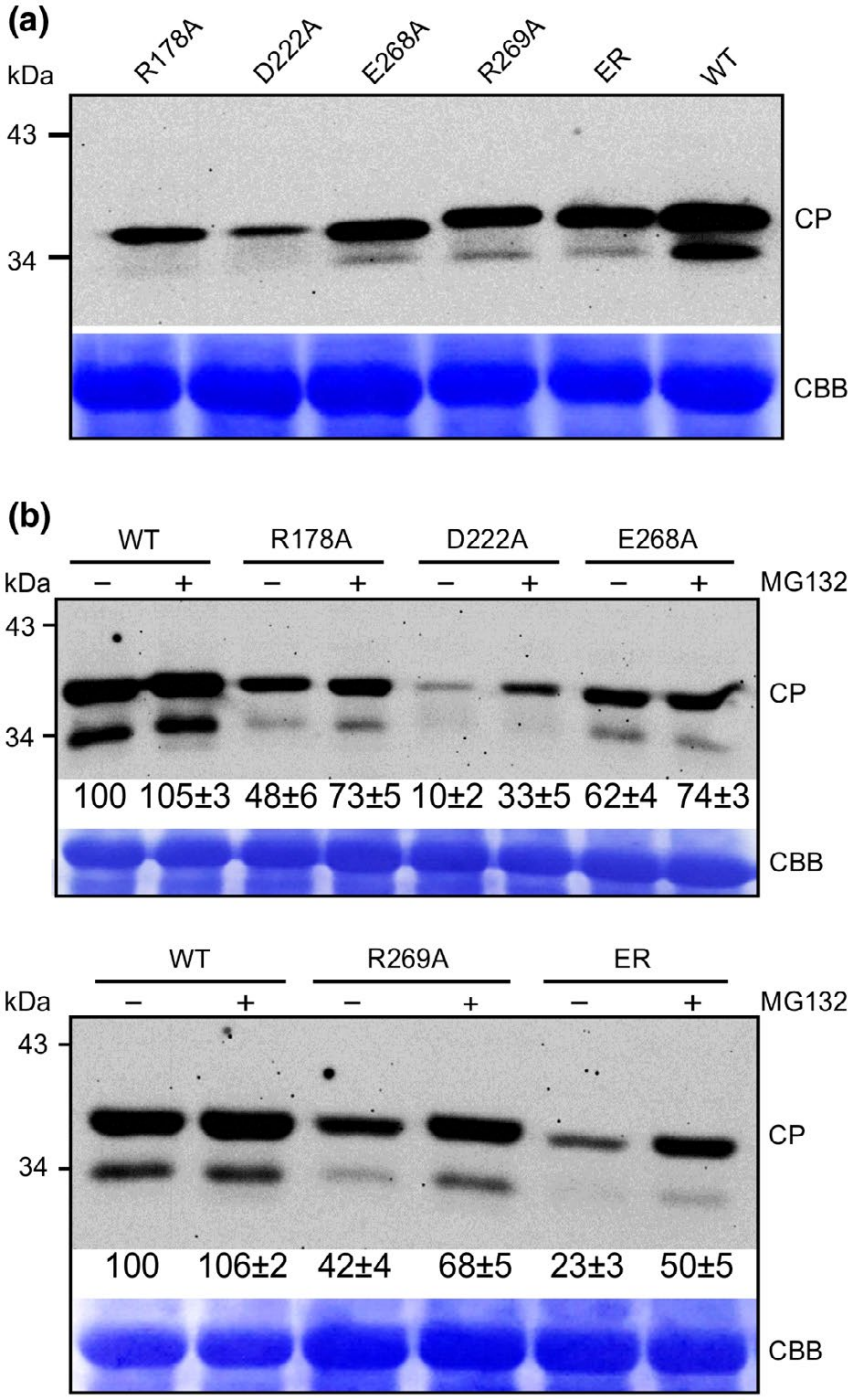
Effects of mutations on the coat protein (CP) stability in *Nicotiana benthamiana* plants. (a) Immunoblotting analysis of transient expression of wild‐type (WT) CP or CPs with point mutation(s). Total protein extracts from inoculated leaf at 48 hr postinoculation (hpi) were probed with TuMV CP antibody. Coomassie brilliant blue staining (CBB) of RuBisCO large subunit serves as a loading control. (b) Effects of the proteasome inhibitor MG132 on the accumulation level of CP. Total protein was extracted from infiltrated patches at 48 hpi. MG132 was infiltrated into *N. benthamiana* leaves 12 hr before harvesting

The fact that the group III mutants R178A and D222A retain replication competency comparable toWT (Figure 4a) but accumulate very low levels of CPs prompted us to hypothesize that the movement‐defective phenotype of the group II and III mutants is associated with the instability of the CP protein. To test this, we conducted a transient expression assay to detect CP accumulation levels. The CP coding sequence of WT and that of the movement‐defective mutants R178A, D222A, E268A, R269A, and ER were cloned into the Gateway binary vector pEarleyGate100, which does not contain a fluorescent protein tag, and the resulting expression vectors were agroinfiltrated into *N. benthamiana* leaves for transient expression. Total proteins were extracted from the agroinfiltrated leaf areas 48 hr postinfection (hpi) for detection of CP by immunoblotting. In comparison with the CP of WT, the CPs of all movement‐defective mutants accumulated at much lower levels (Figure 6a), suggesting a possible link between CP stability and viral intercellular movement.

In plants, most proteins are degraded by the ubiquitin/26S proteasome system, which represents the major protein degradation pathway. To test if this pathway is involved in depleting CP with point mutations, *N. benthamiana* leaves transiently expressing the CPs of movement‐defective mutants were treated with MG132 (carbobenzoxy‐Leu‐Leu‐leucinal), a proteasome inhibitor that is known to block the proteolytic activity of the 26S proteasome complex. Proteins were then isolated, followed by immunoblotting analyses. MG132 treatment effectively increased the CP accumulation levels of R178A, D222A, E268A, R269A, and ER (Figure 6b). These data suggest that the proteasome‐ubiquitin pathway contributes to the CP degradation, and residues R178, D222, E268, and R269 are crucial for CP stability in plants.

### 2.8 CP expressed in *cis* not in *trans* functions in viral cell‐to‐cell and systemic movement

**Figure 7:**
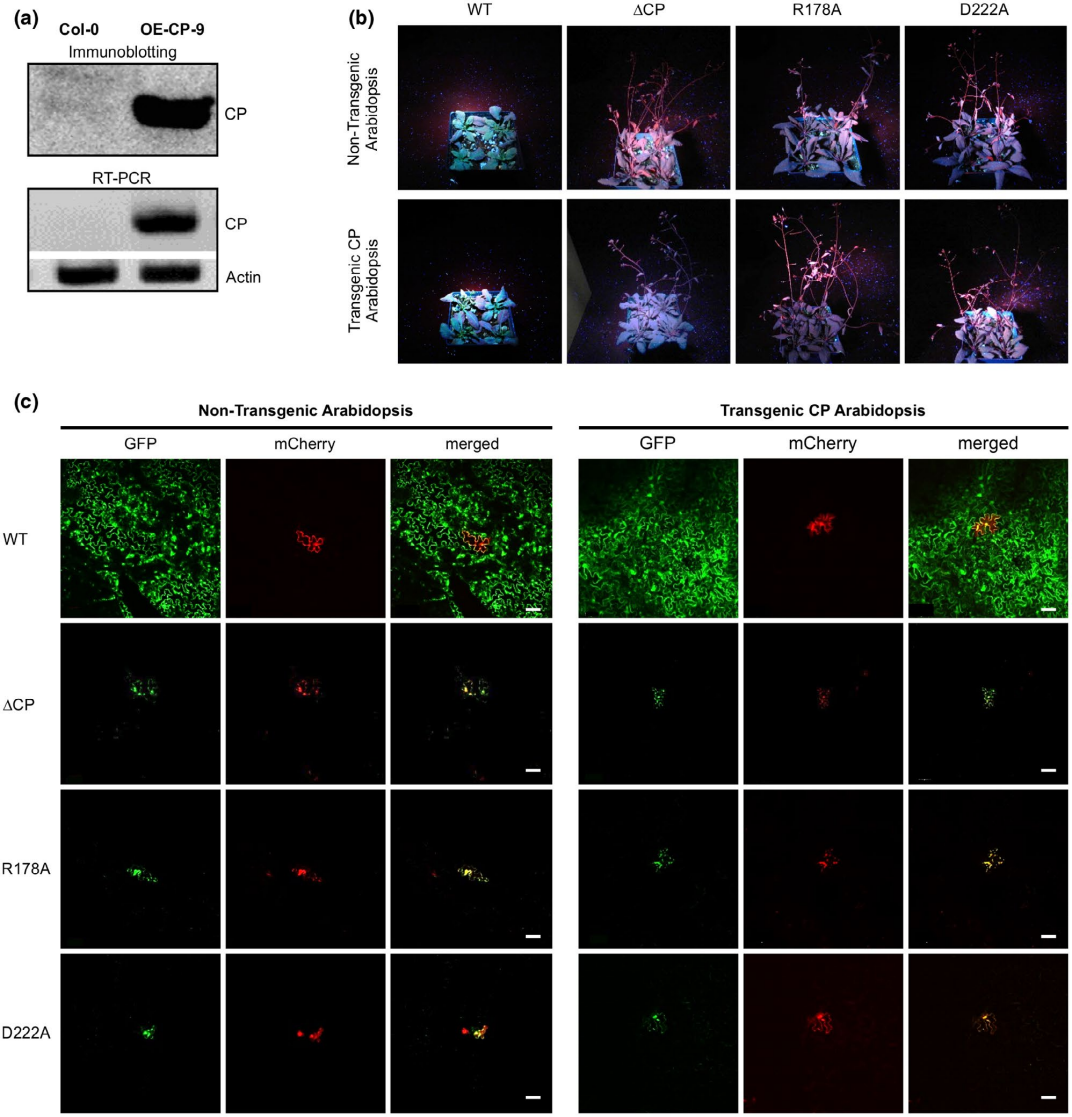
*Trans*‐complementation assay in transgenic *Arabidopsis* plant expressing wild‐type (WT) coat protein (CP). (a) Immunoblotting and reverse transcription (RT)‐PCR analyses of TuMV CP in transgenic overexpression *Arabidopsis* plants. *Actin II* was used as an internal control for RT‐PCR analysis. (b) Analysis of systemic infection of movement‐defective mutants in nontransgenic and transgenic *Arabidopsis* plants by visualization of green fluorescent protein (GFP) fluorescence under UV light at 14 days postinoculation (dpi). (c) Confocal microscopy analysis of cell‐to‐cell movement ability of movement‐defective mutants in nontransgenic and transgenic *Arabidopsis* plants at 6 dpi. Scale bar = 50 μm

To test whether WT CP can rescue the movement‐defect of the mutants ∆CP, R178A, and D222A, we generated transgenic CP *Arabidopsis* lines. Considering that the fluorescent protein tag might negatively affect the CP interactome and virion assembly in plants, we chose the plant expression vector pEarleyGate100 (Early *et al*., 2006), which contains no fluorescent protein tag, to generate the CP expression vector for genetic transformation of *Arabidopsis*. After confirmation of the expression of the TuMV CP by immunoblotting and RT‐PCR (Figure 7a), we observed virus‐like particles (VLPs) with various lengths in those CP‐expressing transgenic plants, consistent with a recent report (Cuesta *et al*., 2019). Three CP transgenic lines and nontransgenic control plants were agroinfiltrated with the WT and the movement‐defective mutants ∆CP, R178A, or D222A (OD_600_ = 0.01). At 14 dpi, either nontransgenic or CP‐transgenic (OE‐CP‐9 as a representative) *Arabidopsis* plants infiltrated with theWT showed typical TuMV symptoms, and under UV light these plants emitted strong green fluorescence. In contrast, infiltration with ∆CP, R178A, or D222A failed to induce systemic infection in both nontransgenic and CP‐transgenic plants (Figure 7b). Similar results were observed using higher infiltration dosages (by increasing OD_600_ value from 0.01 to 0.1 or 1.5). These data suggest that stable expression of the wild‐type CP cannot rescue the mutants ∆CP, R178A, and D222A to establish systemic infection in *Arabidopsis*.

We then determined whether stable overexpression of the wild‐type CP could restore the ability of ∆CP, R178A, and D222A to move intercellularly in the infiltrated leaves. After agroinfiltration of WT virus, ∆CP, R178A, or D222A mutants at a low dosage (OD_600_ = 0.003) into nontransgenic and CP‐transgenic *Arabidopsis* plants, we examined the infiltration area under a confocal microscope at 6 dpi. We observed inoculated cells highlighted by green and red fluorescence as well as large infection foci displaying green fluorescence only emitted by secondary infected cells in the WT‐infiltrated leaves of both nontransgenic and CP‐transgenic *Arabidopsis* (Figure 7c). In nontransgenic and CP‐transgenic *Arabidopsis* plants infiltrated with the mutants ∆CP, R178A, or D222A we detected primary infected cells that emitted green and red fluorescence. However, we could not find any cells emitting only green fluorescence (Figure 7c). Thus, the CP expressed in transgenic plants did not functionally complement ∆CP, R178A, and D222A to move from the primary infection cells to the adjacent cells.

**Figure 8:**
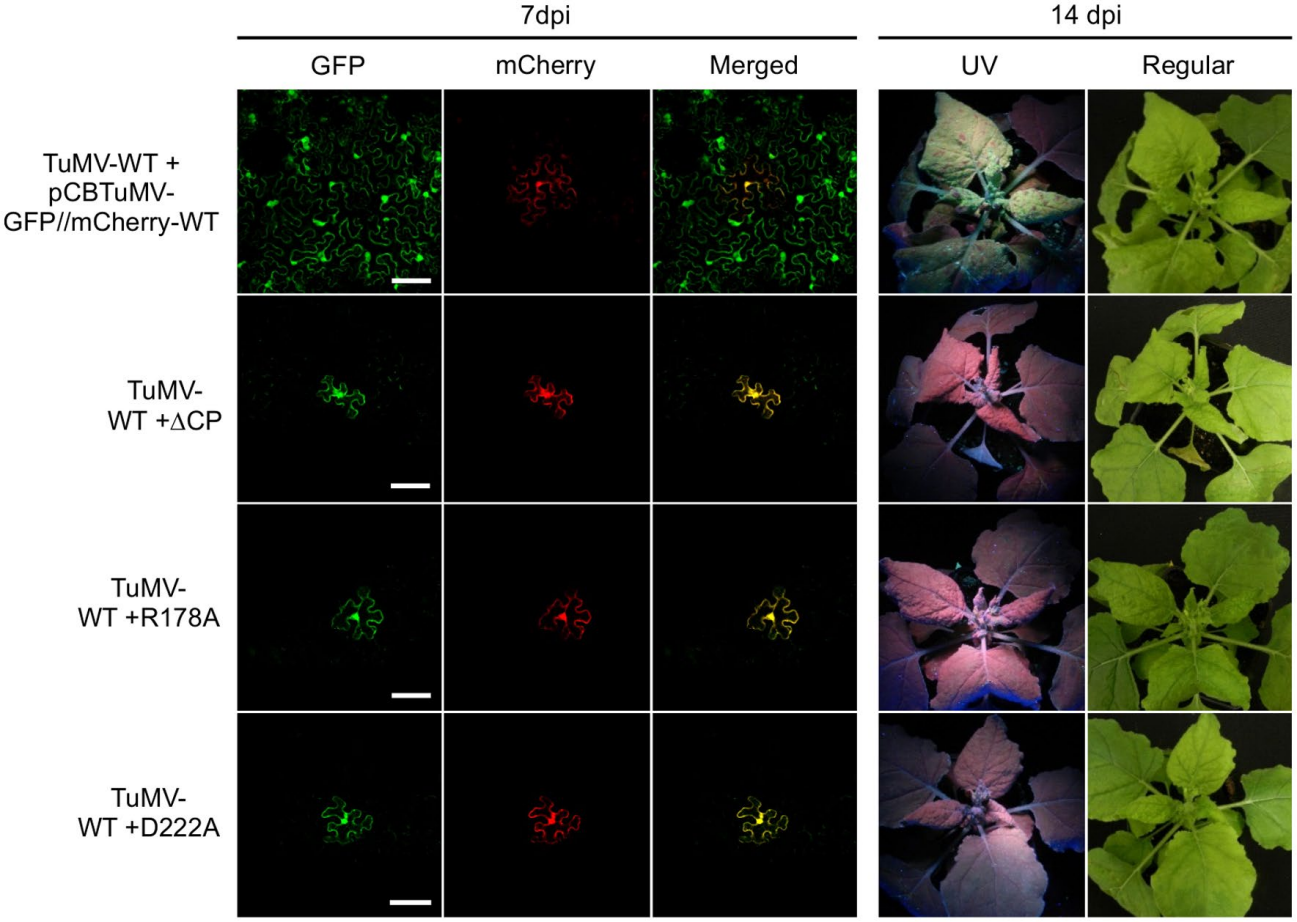
*Trans*‐complementation assay in *Nicotiana benthamiana* plants with wild‐type (WT) TuMV. WT TuMV (without any fluorescent protein tag, TuMV‐WT) was co‐agroinfiltrated with pCBTuMV‐GFP//mCherry‐WT or one of the movement‐defective mutants ΔCP, R178A, and D222A into *N. benthamiana* leaves. Cell‐to‐cell movement of the movement‐defective mutants in the infiltrated leaves was monitored by confocal microscopy and systemic movement of the movement‐defective mutants was determined by visualization of green fluorescent protein (GFP) fluorescence under UV light and reverse transcription‐PCR analyses. Representative confocal images were taken (left panel) at 7 days postinoculation (dpi) and photographs of plants under UV or regular light were taken at 14 dpi. Scale bar in confocal images = 100 μm

To further examine if this also holds true in the context of viral infection, we checked whether the movement‐defective phenotype of the TuMV CP mutants could be rescued by a wild‐type TuMV. A wild‐type TuMV without any fluorescent protein tag (TuMV‐WT) was agroinfiltrated into *N. benthamiana* leaves together with one of the three movement‐defective mutants ΔCP, R178A, or D222A. Confocal microscopy analysis revealed that TuMV‐WT failed to restore the movement defect of the mutants in the inoculated leaves (Figure 8). In these plants, the movement‐defective mutants could not be detected in the upper newly emerged leaves (Figure 8). Taken together these data suggest that the TuMV CP functions in *cis* for viral intercellular movement.

## 3 DISCUSSION

### 3.1 The *cis*‐expression of CP is essential for TuMV translocation in plants

In this report, we present evidence that deletion of TuMV CP or important segments does not affect viral replication but abolishes viral cell‐to‐cell and long‐distance movement in plants (Figure 1). We show that mutations of some charged residues conserved in these segments among potyviruses also result in a similar movement‐defective phenotype (Figure 3). Overall, these data are consistent with recent and previous findings for other potyviruses such as TEV, PVY, and SMV that potyviral CP is required in virus translocation or systemic infection in plants (Seo *et al*., 2013; Kezar *et al*., 2019).

To examine if CP provided in *trans* could rescue the movement defect of TuMV CP deletion or point‐mutation mutants, we generated transgenic *Arabidopsis* plants overexpressing TuMV CP and then inoculated them with the movement‐defective TuMV CP mutants. We confirmed CP expression in these plants (Figure 7), and observed VLPs in these transgenic plants, in agreement with recently published data (Cuesta *et al*., 2019). To our surprise, we found that overexpression of CP failed to rescue these movement‐defective mutants (Figure 7). We further revealed that CP provided by a wild‐type TuMV did not recover the movement defect of all the three CP mutants tested (Figure 8). Altogether these data clearly demonstrate that for TuMV intercellular movements, only the *cis*‐expressed CP is functional. Inconsistent with our results, an earlier study with a recombinant TEV tagged by the reporter protein β‐glucuronidase (TEV‐GUS) showed that overexpression of TEV CP in transgenic *Nicotiana tabacum* plants partially restores the movement defect of four CP mutants: an N‐terminal CP deletion mutant, two single point‐mutation mutants of the charged residues in the core domain, and a double‐mutation mutant of these two residues (Dolja *et al*., 1994). Thus, *trans*‐expressed CP is partially functional for TEV intercellular movement. It is not clear if this difference in the requirement of the *cis* or *trans* expression of CP for viral intercellular movement is due to different potyviruses. It would be interesting to determine which mode is required by other potyviruses and the underlying molecular mechanism.

### 3.2 The role of the N‐terminal domain of TuMV CP in viral translocation and virion assembly

The N‐terminal domain of potyviral CPs exposed on the surface of virus particles is highly variable among potyviruses and contains the DAG motif that is required for aphid transmission (Allison *et al*., 1985; Shukla *et al*., 1988; Dolja *et al*., 1991; López‐Moya *et al*., 1999; Nigam *et al*., 2019). In this study, we found that the N‐terminal segment amino acids 6–50 of the N‐terminal domain of TuMV CP was dispensable for viral cell‐to‐cell and long‐distance movement, as deletion of this region did not significantly affect viral movement in *N. benthamiana* leaf (Figure 1). The Δ6–50 mutant produced 95% typical TuMV virions and 5% aberrant particles with an average length of 1,500 nm (Figure 2b). As the N‐terminal 50 amino acids was shown also to have the self‐interaction ability (Figure 2c), we speculate that the N‐terminal domain might play a role in virion maturation and/or termination of virion formation.

Our data are consistent with a study with ZYMV demonstrating that deletion of the N‐terminal 33 amino acids in the N‐terminal domain of CP does not affect viral infection in either the inoculated or upper leaves (Arazi *et al*., 2001). However, our findings are different from several other studies. In an early report, a TEV CP‐truncated mutant lacking the N‐terminal amino acids 5 to 29 exhibits slow cell‐to‐cell movement and fails to establish systemic infection (Dolja *et al*., 1994). In a recent study, deletion of the N‐terminal 50 amino acids of CP in the PVY genome reduces viral infectivity in the inoculated leaves and abolishes the ability to establish systemic infection (Kezar *et al*., 2019). Different from these, the N‐terminal segment amino acids 6 to 27 of WSMV CP is not essential for long‐distance movement but is required for efficient cell‐to‐cell movement (Tatineni and French, 2014; Tatineni *et al*., 2014). It seems that the requirement of the variable N domain (at least its N‐terminal segment) for cell‐to‐cell movement and systemic infection of the viruses in the *Potyviridae* family may vary from virus to virus. It is worth mentioning that the atomic structure‐based definition of the N, core, and C domains of potyviral CPs was not done until recently (Zamora *et al*., 2017; Cuesta *et al*., 2019; Kezar *et al*., 2019). Previously, the C‐terminal segment of the N domain was included in the core domain. A functional analysis of this segment is yet to be done for a better understanding of the involvement of the N domain in potyviral virion assembly and intercellular movement.

### 3.3 The role of the core domain of TuMV CP in viral intercellular movement and virion assembly

In this study, we revealed that alanine substitution of charged residues R178 and D222 in the core domain abolished viral cell‐to‐cell movement in *N. benthamiana* plants (Figure 4d) and no virions were detected in *N. benthamiana* protoplasts transfected with these two point mutants (Figure 5b), suggesting that Arg at 178 and Asp at 222 are critical for TuMV virion assembly and intercellular movement. Similar results have been observed in the corresponding analogous point mutants of a few other potyvirid species, including TEV (Dolja *et al*., 1994) and WSMV (Tatineni *et al*., 2014). It is tempting to speculate that there is a correlation between virion assembly and potyviral intercellular movement. This suggestion contrasts against the assumption made by Grangeon and colleagues that TuMV moves as VRCs for cell‐to‐cell movement, based on their observation that the motile 6K2‐containing vesicles (VRCs) enable vRNA transport to PD by trafficking along ER/microfilaments and pass through the PD to the neighbouring cells (Grangeon *et al*., 2012, 2013). However, our data cannot exclude the possibility that CP expressed in *cis* also supports TuMV and related viruses to move intercellularly as RNPs.

It has been suggested that positively charged residue R178 may interact with negatively charged residues D222 via a salt bridge, which may be critical for protein stability, virion assembly, and viral cell‐to‐cell movement (Dolja *et al*., 1991). To test this hypothesis, we created a TuMV CP double mutant (DR) in which Arg178 and Asp222 were switched. Similar to the single mutants R178D and D222R, the double mutant DR failed to move in *N. benthamiana* plants (Figure 4d). As structural changes might occur after switching the two residues, our data could not completely reject the salt bridge hypothesis.

We speculated that the charged residues R178 and D222 might be important for proper folding on translation to form the appropriate functional three‐dimensional structure and thus mutation of these two residues might affect CP stability. Indeed, immunoblotting analyses revealed that the CP accumulation levels of the mutants R178A and D222A in *N. benthamiana* leaves were remarkably reduced compared to that of WT CP (Figure 6a). Treatment with the proteasome inhibitor MG132 could partially inhibit R178 and D222 CP degradation (Figure 6b), suggesting that these charged residues at least partially contribute to CP stability, which unavoidably affects CP functionality. In a recent study, Gallo and colleagues revealed that the formation of stable PPV virions requires CP and other viral factors such as HC‐Pro and a replication‐proficient RNA (Gallo *et al*., 2018). Their data suggest a functional link between RNA replication and virion assembly. Recent near‐atomic structural analyses of PVY and TuMV virions and VLPs strongly suggest that CP–RNA interaction is crucial for the helical configuration and stability of the virion (Cuesta *et al*., 2019; Kezar *et al*., 2019). Therefore, it is likely that TuMV virion assembly requires CP expression and viral replication in a tightly coordinated manner.

### 3.4 The role of the C domain of TuMV CP in viral cell‐to‐cell movement

The segment of amino acids 265–274 of the C domain of TuMV CP is required for viral intercellular spread as deletion of this domain abolished viral cell‐to‐cell and systemic movement (Figure 1, Table 2). Moreover, a single point mutation (E268A or R269A) in this domain or the double mutation of these two residues (ER) is sufficient to partially compromise the intercellular movement of TuMV, leading to a significant delay in the onset of systemic symptoms (Figures 3 and 4, and Tables 1 and 2). Consistent results have been observed in several other potyviruses. For example, it has been shown that the deletion of the C‐terminal region of either PVY CP or TEV CP and point mutations of important charged amino acids in the C‐terminal region of SMV CP disrupted viral intercellular movement (Dolja *et al*., 1995; Seo *et al*., 2013; Kezar *et al*., 2019). Therefore, the C domain of potyviral CPs is crucial for viral cell‐to‐cell and long‐distance movement.

**Table 2:** Summary of the phenotypes of coat protein (CP) mutants in *Nicotiana benthamiana*

Mutants	Mutation location^a^	Replication^b^	Cell‐to‐cell movement^c^	Systemic movement^d^	Assembly^e^
ΔCP		+	−	−	NT
Δ6‐50	N	+	+	+	+
Δ51‐199	N + Core	+	−	−	NT
Δ200‐283	Core + C	+	−	−	NT
Δ265‐274	C	+	−	−	NT
R178A	Core, Helix 5	+	−	−	−
R178D	Core, Helix 5	+	−	−	NT
R195A	Core, Helix 6	+	+	+	+
D222A	Core, Bend	+	−	−	−
D222R	Core, Bend	+	−	−	NT
DR	Core, Helix 5 + Bend	+	−	−	NT
D257A	C, NS	+	+	+	+
E265A	C, NS	+	+	+	+
E268A	C, NS	+	Slow	Slow	+
R269A	C, NS	+	Slow	Slow	+
ER	C, NS	+	Slow	Slow	+
D274A	C, NS	+	+	+	+

^a^ Mutation location is determined based on the atomic model of TuMV CP (PDB: 6T34). N, N‐ terminus; Core, core domain; C, C‐terminus; NS, no secondary structure assigned.

^b^ Determined by the protoplasts transfection assay. +, no significant difference with the wild‐type (WT) virus.

^c^ Determined by confocal microscopy. +, similar with the WT virus; −, no cell‐to‐cell movement observed; Slow, remarkably slow cell‐to‐cell movement.

^d^ Determined by symptom appearance, UV light, and reverse transcription PCR. +, similar with the WT virus; −, no systemic infection observed; Slow, delayed long‐distance movement.

^e^ Determined by transmission electron microscopy from inoculated leaf samples or transfected protoplasts samples. +, virions observed; −, no virion observed; NT, not tested.

How does the C domain function to support potyviral intercellular movement? Previous studies suggested that the C‐terminus of potyviral CP is exposed on the virion surface and possesses a disordered short segment at the very end (Shukla *et al*., 1988; Zamora *et al*., 2017). Opposed to this, a recent study has determined the near‐atomic structure of PVY virions and revealed that the C domain of PVY CP is completely buried in the lumen of the viral filament, forming a compact conical structure (Kezar *et al*., 2019). The C‐terminal region of SMV CP was shown to be involved in CP intersubunit interactions, implying a possible role of this domain in virion assembly (Kang *et al*., 2006; Seo *et al*., 2013). However, TEV and PVY could form virions without the C‐terminal domain (Dolja *et al*., 1995; Kezar *et al*., 2019), indicating that this domain is not necessary for virion assembly. In this study, we found that the C‐terminal region (including the C domain) of TuMV CP did not self‐interact (Figure 2c). It is possible that the C domain of TuMV CP is required for CP–vRNA binding, as suggested in two independent cryoelectron microscopy structure studies of WMV and PVY virions (Zamora *et al*., 2017; Kezar *et al*., 2019). Alternatively, the C domain of TuMV CP may serve as a site for the CP to interact with other key players (such as host factors) to regulate TuMV cell‐to‐cell and systemic movement. It is of great interest to isolate such factors and characterize their functional roles in viral intercellular movement.

## 4 EXPERIMENTAL PROCEDURES

### 4.1 Plant materials, genetic transformation, and protoplast work

*N. benthamiana* and *Arabidopsis thaliana* ecotype Col‐0 plants used for agroinfiltration were prepared as described previously (Cheng *et al*., 2017; Li *et al*., 2018). In brief, plants were grown in pots with Pro‐Mix Mycorrhizae Growing medium under greenhouse conditions with a 16 hr light/8 hr dark regime. The relative humidity was set at 60% and temperatures were adjusted to 24 and 22°C during the light and dark periods, respectively.

To generate *Arabidopsis* transgenic lines expressing TuMV CP, the coding region of TuMV CP was cloned into the plant expression vector pEarleyGate100 (Early *et al*., 2006) using Gateway technology, and genetic transformation and subsequent screening for transgenic lines were carried out as described (Li and Wang, 2018) for genetic transformation of *Arabidopsis*. Mesophyll protoplasts were prepared from 4‐week‐old *N. benthamiana* leaves following procedures published previously (Yoo *et al*., 2007; Wu *et al*., 2009). Transfection assay was carried out essentially as described previously (Deng *et al*., 2015; Wu *et al*., 2020).

### 4.2 Construction of double fluorescent protein‐tagged TuMV infectious clone pCBTuMV‐GFP//mCherry and CP mutants

The TuMV infectious clone pCamibaTunos/GFP (pCamTuMV‐GFP) and the mini‐binary vector pCB301 were reported previously (Xiang *et al*., 1999; Cotton *et al*., 2009). The GFP‐tagged TuMV infectious clone pCBTuMV‐GFP was constructed by double digestion of pCambiaTunos/GFP with *Xma*I and *Apa*I, and ligation of the TuMV‐GFP fragment into the corresponding sites of pCB301. Using a PPV infectious clone tagged with GFP and mCherry‐HDEL (Cui and Wang, 2016) as a template, we amplified the 35S‐mCherry‐HDEL‐Nos fragment and introduced an *Apa*I digestion site to this fragment by PCR. The amplified fragment was digested with *Apa*I and inserted into the *Apa*I site of pCBTuMV‐GFP to generate the TuMV infectious clone pCBTuMV‐GFP//mCherry. All the vectors were confirmed by DNA sequencing.

To create CP mutants, the CP coding fragment (1,342 bp cDNA between *Mlu*I and *Sal*I) was amplified by PCR from the parent plasmid pCamTuMV‐GFP using Phusion High‐Fidelity DNA Polymerase (New England Biolabs Inc.). The PCR product was then ligated into the pCRBlunt vector (Invitrogen), resulting a recombinant plasmid named pCRBlunt‐CP1342, which was used for deletion or introduction of single‐point or double mutations. Then, the resulting intermediate clones were digested with *Mlu*I and *Sal*I and ligated into the corresponding sites of pCamTuMV‐GFP or pCBTuMV‐GFP//mCherry. CP deletion mutants were generated by overlap extension PCR using appropriate primers, and 12 CP point mutants were constructed by site‐directed mutagenesis using appropriate primers. Restriction enzymes and T4 DNA ligase were purchased from New England Biolabs Inc.

### 4.3 Cell‐to‐cell movement of TuMV CP mutants

Agrobacterial cells harbouring TuMV CP mutants were inoculated into 3‐ to 4‐week‐old *N. benthamiana* plants at OD_600_ = 0.0001. The cell‐to‐cell movement ability of mutants was determined by confocal microscopy by monitoring the green fluorescent foci on the agroinfiltrated *N. benthamiana* leaf using a TCS SP2 confocal laser scanning microscope (Leica) starting at 2 dpi.

For *trans*‐complementation assay in transgenic *Arabidopsis* lines, 3‐week‐old *Arabidopsis* plants expressing TuMV CP were agroinfiltrated with movement‐defective TuMV mutants at an OD_600_ value of 0.003 and cell‐to‐cell movement was monitored on the agroinfiltrated leaf area using a TCS SP2 confocal laser scanning microscope starting at 4 dpi and continually monitored each day until 10 dpi. Long‐distance movement was evaluated by visual observation for symptoms, green fluorescence under UV light, and RT‐PCR. Three independent experiments were performed and each trial included at least five individual plants.

For *trans*‐complementation assay with WT TuMV, two agrobacterial cultures harbouring TuMV‐WT (tag‐free) and one of the three mutants ΔCP, R178A, or D222A were mixed in three different ratios, 100:1, 10:1, and 1:1 (vol/vol), and the mixed cultures were infiltrated into levels of 3‐ to 4‐week‐old *N. benthamiana* plants. Coinfiltration of TuMV‐WT and pCBTuMV‐GFP//mCherry‐WT served as the control. Cell‐to‐cell and long‐distance movement of the mutants were evaluated as described above.

### 4.4 Crude virion preparation and electron microscopy

To obtain the crude virion preparation from *N. benthamiana* leaf tissues, 0.1 g of leaves with symptoms were ground in a mortar and pestle with 150 µl of potassium phosphate buffer (pH 7.0) containing 0.1% β‐mercaptoethanol. Following the centrifugation at 12,000 × g for 5 min at 4°C, the supernatant was filtered through a 40 µm Nylon Cell Strainer (Corning Inc.). Fifty microlitres of chloroform were then added and vortexed for 1 min, the homogenate was then centrifuged at 12,000 × g for 10 min at 4°C. The aqueous phase was collected and subjected to centrifugation at 12,000 × g for 30 min at 4°C. The resulting aqueous phase was used for negative staining. For crude virion preparation of *N. benthamiana* mesophyll protoplasts, protoplasts were harvest at 72 hpt by centrifugation at 50 × g for 2 min. Fifty microlitres of potassium phosphate buffer (pH 7.0) were added to the pellet, followed by the procedure presented above. Formvar/carbon‐coated EM grids (Electron Microscopy Sciences) were incubated with the crude virion preparation for 2 min and stained with 2% phosphotungstic acid (PTA), pH 7.0, for 2 min. Grids were allowed to dry prior examination on a transmission electron microscope (JEM‐1200EXII, JEOL Ltd) operated at 80 kV.

### 4.5 RNA extraction and RT‐qPCR

Total RNA was extracted from leaf tissues or protoplasts using the Plant Total RNA Mini Kit (Geneaid) as instructed. For first‐strand cDNA synthesis, 1 μg of RNA was pretreated by DNase I (Invitrogen) at 37°C for 20 min. The reverse transcription reaction was performed using a SuperScript III First‐Strand Synthesis System (Thermo Scientific) following the manufacturer's instructions. RT‐qPCR analyses of TuMV RNA levels were performed as described previously (Wu *et al*., 2020).

### 4.6 Y2H and BiFC assays

Y2H and BiFC assay were essentially as described (Li *et al*., 2018). Genes of interest were cloned into Y2H Gateway vectors pDEST‐GBKT7 and pDEST‐GADT7 or BiFC Gateway vectors p35S‐YN and p35S‐YC (Lu *et al*., 2010).

### 4.7 Protein work

Immunoblotting and relative quantification of proteins were performed as described (Cheng and Wang, 2017).

### 4.8 Chemical treatment

Chemical treatment with the proteasome inhibitor MG132 was performed essentially as described (Cheng and Wang, 2017). In brief, 100 μM MG132 (Sigma‐Aldrich) was infiltrated into *N. benthamiana* leaves for 12 hr before harvesting.

## Data Availability

The data that support the findings of this study are available from the corresponding author upon reasonable request.
